# Photo-realistic traffic scene datasets: real and synthetic perspectives for vision-based analysis

**DOI:** 10.1016/j.dib.2025.111976

**Published:** 2025-08-12

**Authors:** Khulan Khalzaa, Stephen Karungaru, Kenji Terada, Tsend-Ayush Chimed-Ochir

**Affiliations:** a B1 Research Laboratory, Department of Computer Science, Tokushima University, Tokushima 770-8502, Japan; b School of Information and Communication Technology, Mongolian University of Science and Technology, Ulaanbaatar 13341, Mongolia

**Keywords:** Data generation, Real and synthetic data, Road users, Scene understanding, Traffic scene

## Abstract

Perception plays a crucial role in autonomous driving and computer vision, particularly in interpreting traffic scenes from monocular cameras. In this article, we present a comprehensive collection of traffic scene datasets organized into four distinct groups: (1) Traffic Scene Datasets, (2) Top-View Datasets - both introduced in the authors’ earlier research, (3) MultiHeightView Datasets and (4) Depth Datasets. The Traffic Scene Datasets include RealStreet, which captures authentic traffic scenarios, and SynthStreet, its synthetic counterpart. To enhance the diversity of available data, we also introduce the SupporterReal subset, which augments real datasets that lack a corresponding synthetic twin. In addition, SupporterVirtual offers a virtual environment without a direct real-world equivalent. For top-view perspectives, the datasets RealTop and SynthTop provide real and synthetic traffic scene data, respectively.

The MultiHeightView dataset provides synchronized RGB image sequences of urban traffic scenes captured from three fixed camera heights: 0.5 m, 1.0 m, and 1.5 m designed to support zero-shot monocular depth model. Depth dataset provides synchronized RGB, and depth images of real-world urban traffic scenes collected using RealSense stereo camera. A perceptual similarity analysis using the LPIPS metric confirms that synthetic images closely resemble real-world scenes, supporting their use in synthetic-to-real domain transfer learning. Data collection and generation were conducted between 2023 and 2025, utilizing standardized formats to ensure compatibility with deep learning frameworks and to promote broader reuse in computer vision applications.

Specifications Table

**Table:** 

Subject	Computer Vision and Pattern Recognition.
Specific subject area	Traffic scene analysis within the broader fields of autonomous driving and computer vision*.*
Type of data	Raw and generated data including images and videos with supporting materials (.json, .npy and .txt formats).
Data collection	Videos in this dataset were recorded with a COOAU D30 1080P dash camera mounted in a car, an iPhone 15 Pro Max on a tripod, and RealSense D457 camera on a tripod, capturing urban traffic scenes. Each video clip has a duration of 10-20 seconds and is recorded in 1920 × 1080 resolution. The dataset selection includes scenes featuring pedestrians, cyclists, and vehicles in urban traffic environments*.*
Data source location	Country: Tokushima, Japan.The data collection took place at the following locations: 1. 3 Chōme Tokushimachō, Tokushima, 770-0852, Japan (Latitude: 34°04′31.6"N, Longitude: 134°33′26.4"E) 2. 2 Chōme Tokushimachō, Tokushima, 770-0852, Japan (Latitude: 34°04′27.3"N, Longitude: 134°33′25.9"E) 3. 6 Chōme Yoshinohonchō, Tokushima, 770-0802, Japan, (Latitude: 34°05′10.5"N, Longitude: 134°33′07.5"E) 4. 3 Chōme Tokushimachō, Tokushima, 770-0852, Japan (Latitude: 34°04′31.3"N, Longitude 134°33′26.8"E)
Data accessibility	Repository name: Photo-Realistic Traffic Scene Datasets: Real and Synthetic Perspectives Data identification number: doi: 10.17632/rynrzt2f6t.1 Direct URL to data: https://data.mendeley.com/datasets/rynrzt2f6t/1 Instructions for accessing these data: None
Related research article	K. Khalzaa, S. Karungaru and K. Terada, ``Creation and Testing of Synthetic Datasets for Training Road Scenes Algorithms,'' 2023 32nd IEEE International Conference on Robot and Human Interactive Communication (RO-MAN), Busan, Korea, Republic of, 2023, pp. 1587-1592, doi: 10.1109/RO-MAN57019.2023.10309307 [1].

## 1 Value of the Data

• The dataset provides a comprehensive collection of real-world and synthetic traffic scenes, including horizontal view, top-view, multi-height perspectives, and depth related data, serving as a valuable resource for urban traffic analysis.
• Its diverse data types support the development and evaluation of perception models for traffic monitoring, road user detection, semantic segmentation, depth estimation and traffic scene understanding.
• The approach encourages synthetic data utilization in regions with privacy restrictions, such as Japan, where public video recording is legally and culturally constrained. It demonstrates how limited real-world data can be augmented with photorealistic synthetic scenes for research and model training.
• Synthetic datasets, generated using Lumion [7], provide a controlled environment for simulating diverse urban scenarios, lighting conditions, and camera perspectives. This allows researchers to expand scarce real-world datasets using scalable synthetic data, improving model robustness and generalization.
• The dataset enables synthetic-to-real domain transfer learning, by providing paired real and synthetic scenes under consistent conditions. This is critical for studying generalization, robustness, and adaptation of perception models.
• It provides a scalable and reproducible resource for training and evaluating machine learning models in privacy-constrained environments, helping to bridge the gap between limited real-world data and the need for large-scale annotated datasets.

## 2 Background

Privacy concerns are a critical consideration in the collection of images and videos in public spaces. The challenge of data scarcity, coupled with the need for controlled environments, makes obtaining diverse and representative real-world datasets difficult. Synthetic data provides a structured and customizable alternative, ensuring compliance with privacy regulations while supplementing real-world data.

Traffic scene analysis has benefited from datasets such as KITTI [2], Virtual KITTI (vKITTI) [3], and CARLA [4]. While many synthetic datasets rely on 3D engines like Unity3D [5] or video game environments such as GTA [6], this dataset utilizes Lumion, a photorealistic 3D rendering software. By leveraging Lumion, this dataset aims to provide high-quality synthetic traffic scenes that can complement real-world data.

Unlike existing datasets developed by separate research groups, this collection provides both real-world and synthetic datasets under a single authorship. This unified approach offers a cohesive resource for the research community, supporting applications in traffic scene analysis, road user detection, and depth estimation*.*

## 3 Data Description

**Fig. 1:**
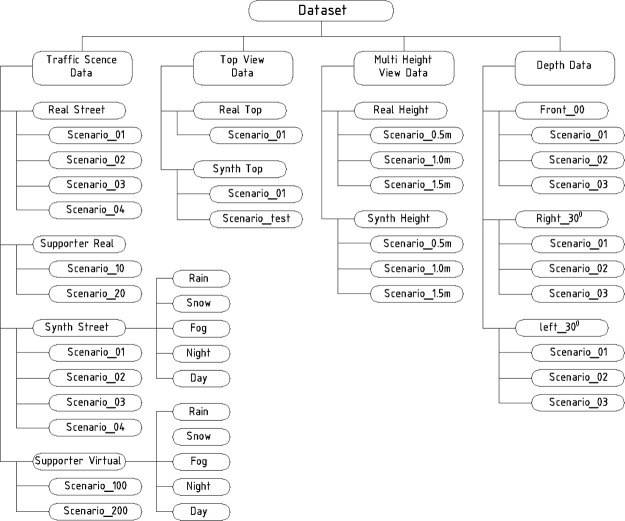
Dataset structure and its categories.

The dataset is structured into several main directories, each containing specific types of data relevant to urban traffic scene analysis. As illustrated in Fig. 1, this hierarchical organization categorizes data into real and synthetic subsets, facilitating clear separation and efficient navigation*.*

**1. Traffic Scene Data:** This dataset was first introduced in our earlier work [1]. In the present paper, we provide an expanded and more detailed account of the collection process, including additional technical parameters and configuration settings to support reproducibility for the first public release.

**RealStreet**: Dataset captures authentic road-user interactions in urban traffic environments. The data was organized into four directories based on day and diverse location, stored in JPEG format with accompanying metadata files. Two scenarios were captured while the car was stationary at an intersection, and the other two were recorded while the car was in motion. The dataset includes the following typical urban traffic contexts:

• Multi-lane intersections with vehicle flows, pedestrian crosswalks, and traffic lights.
• Narrow residential or mixed-use roads, often featuring parked vehicles, sidewalks, and cyclists sharing the road.
• Main arterial roads with clear lane markings, signage, and roadside infrastructure such as poles, curbs, and greenery.

**Fig. 2:**
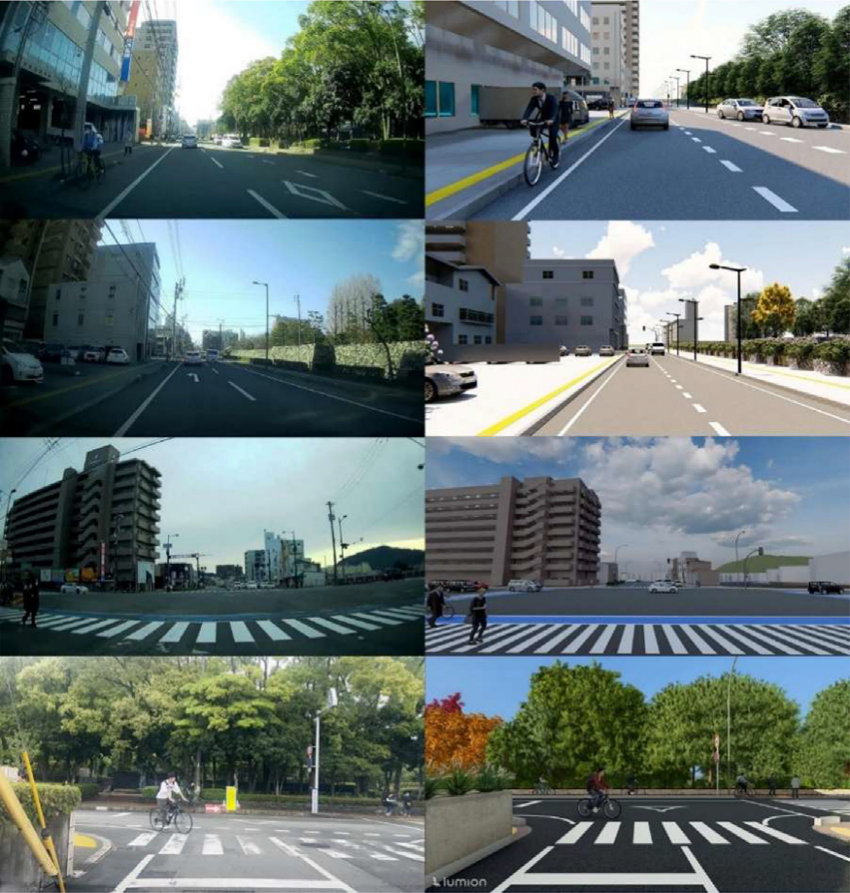
Sample frames of RealStreet and SynthStreet adapted from [1].

Presence of vulnerable road users (VRUs) such as pedestrians and cyclists in various positions and orientations, enhancing the diversity of behavior and spatial relations in the dataset. Examples of traffic scenes captured from both real and synthetic environments, showcasing a range of urban road configurations and user behaviors, are presented in Fig. 2. The first three rows are adapted from our previous work [1], and a new fourth scenario has been added in this release to extend scene diversity. The RealStreet dataset contains a total of 960 images, with 240 images collected for each of the four scenarios. Videos are recorded at full HD resolution with a duration of 10 seconds per clip. The dataset included three classes for 2D object detection. 2D bounding box annotations are provided for each image, indicating the locations of vehicles, cyclists and pedestrians within the scenes.

**SupporterReal**: Dataset provides supplementary real-world traffic scenes that do not have a synthetic counterpart, broadening the variety of intersection types and traffic patterns available for training. It contains two scenarios, a total of 480 images. Unlike other datasets, SupporterReal has no synthetic counterpart.

**Fig. 3:**
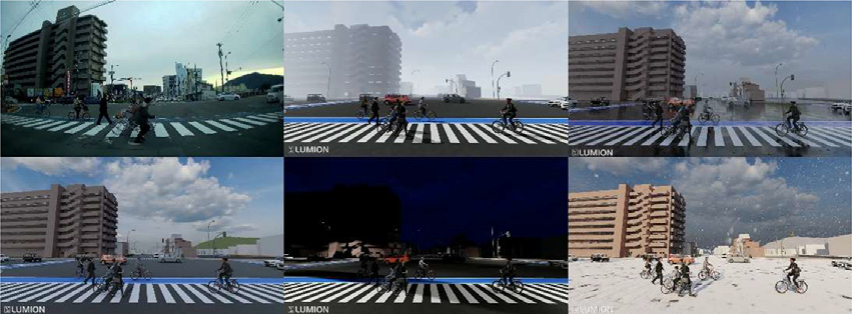
Weather variation simulations used to enrich synthetic datasets, adapted from [1].

**SynthStreet**: The SynthStreet dataset was created by reproducing all four RealStreet scenarios in a synthetic format. Scenes were modeled and rendered to match the layouts, viewpoints, and traffic conditions of RealStreet, enabling controlled training and evaluation without the privacy constraints of real-world data. For each scenario, five environmental variations were generated: daytime, nighttime, rain, fog, and snow, thus we obtained five screenplays per scenario (See Fig. 3). Unlike RealStreet, where each scenario contains only 240 real images, SynthStreet expands this number to 1,200 images (240 images per condition × 5 conditions) to account for variations in weather conditions. In total, the dataset includes 4800 images with 2D bounding box annotations, which precisely delineate object boundaries to facilitate detailed analysis. Initially, the dataset included three classes for object detection and segmentation in 2023 [1]. This release now features 11 classes for object detection and 13 classes with both semantic and instance segmentation annotations.

**SupporterVirtual**: A virtual environment supporting SynthStreet was developed without a real-world counterpart, serving as a supplementary resource for generating diverse traffic scenarios. Supporter Virtual comprises two entirely artificial street layouts developed to expand the synthetic data collection. Each of these virtual streets consists of 10-second videos, 240 images. Both 2D bounding box and 2D segmentation annotations are provided for SupporterVirtual dataset. Then the SupporterVirtual dataset was rendered under various weather conditions and augmented, resulting in fivefold increased in the number of images.

While the concept of SupporterReal and SupporterVirtual was first introduced in [1], the current release offers standardized formatting and metadata for integration with the other datasets.

**2. Top-View Data:** The top-view dataset was previously referred to as *Synth-Aerial* and *Real-Aerial* in our earlier work [8]. In this release, the names have been updated to better reflect the top-view perspective and to align with our dataset naming conventions. This version constitutes the first public release of the dataset, which has updated annotations, and standardized formatting. It has been developed specifically for the detection, and speed estimation of vulnerable road users (VRUs).

**RealTop**: Dataset was developed in response to UAV usage restrictions in many urban areas, including Japan. High-resolution overhead imagery of urban traffic scenes was acquired using cameras mounted on elevated platforms, to achieve a true top-down perspective. This top-down perspective enables detailed observation of VRU and vehicle trajectories. Each video segment is recorded at 30 frames per second to support real-time processing and spans 18 seconds, yielding around 500 annotated frames. Cameras were positioned roughly 20–30 m above the roadway, producing near-vertical views of intersections. Bounding boxes were drawn for all visible road users from the overhead perspective.

**Fig. 4:**
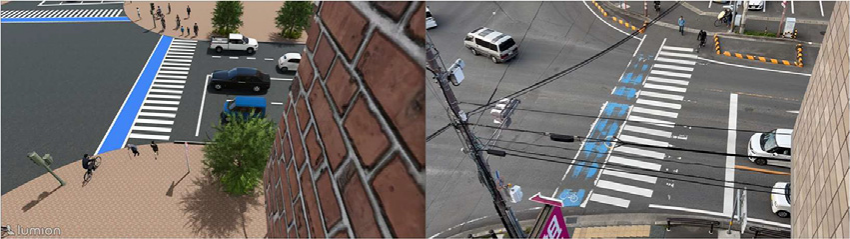
Samples of top-view data, adapted from [8].

**SynthTop**: The synthetic top-view datasets replicate real-world overhead perspectives by reproducing the spatial layout and camera geometry of RealTop. It recreates RealTop viewpoints within a simulated Lumion environment, allowing precise control over traffic density, VRU behavior, and camera settings. As illustrated in Fig. 4, synthetic scenes generated in Lumion (left) are compared with real top-view photographs (right), showing intersection layouts and VRU movement patterns. This extends the top-view scenarios first introduced in our prior work [8]. Through augmentation, each 18-second clip yields about 660 frames. A dedicated test scenario is included for evaluation.

**3. MultiHeightView Data:** This height-based RGB subset was designed to support Monocular depth model evaluation under varied vertical viewpoints and viewpoint sensitivity analysis.

**RealHeightView**: The dataset contains synchronized image pairs captured at three heights to address challenges caused by occlusion and low-angle perspectives: 0.5 m, 1.0 m, and 1.5 m. Each video at these heights has a duration of 15 seconds and contains 360 images, resulting in a total of 1,080 images across the three heights.

**Fig. 5:**
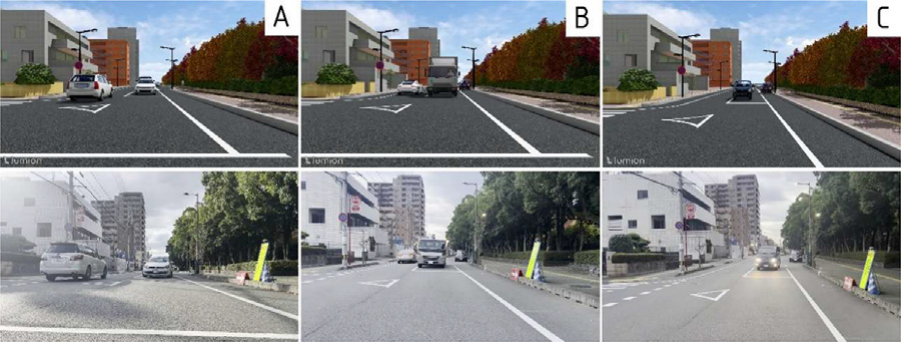
Samples of MultiHeightView data.

**SynthHeightView**: As shown in Fig. 5, synthetic height view data were generated using Lumion at three fixed camera heights: 0.5 m (Section A), 1.0 m (Section B), and 1.5 m (Section C). To maintain consistency with the RealDepth dataset, a total of 1,080 images were generated, 360 per height category.

**4. Depth Data:** This dataset contains synchronized RGB and depth recordings of real-world outdoor traffic scenes, collected using the Intel RealSense D457 camera.

**Fig. 6:**
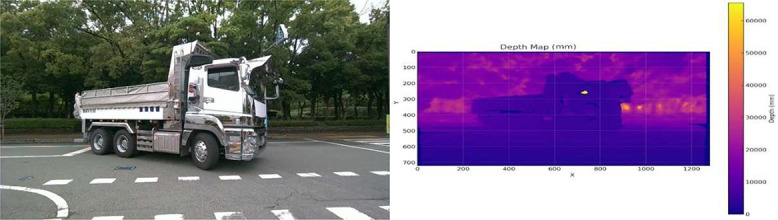
Example of a synchronized RGB image (left) and its corresponding depth map (right) captured using the Intel RealSense D457 stereo camera.

The depth values were exported as .npy files and visualized using colormaps for qualitative inspection. As shown in Fig. 6, the depth map preserves the scene’s spatial structure, including large vehicles such as trucks, which are critical for traffic scene understanding. Although the D457 supports depth estimation up to 20 meters, its reliable operational range is within 6 meters, beyond which the depth accuracy diminishes due to stereo disparity limitations and outdoor lighting conditions. This sensor limitation was shown in Fig. 6 as evident in the far background regions, where the depth values showed increased noise or saturation (e.g., at 65535 mm). The mean depth across sampled frames was approximately 11.1 meters, but the most confident measurements are located within the foreground range (<6 meters).

## 4 Experimental Design, Materials and Methods

The data acquisition process involved both real-world data collection and synthetic data generation, employing various tools, instruments, and software for image capture, annotation, and dataset creation. While the detailed process of generating the Traffic Scene Dataset was outlined in our previous work [1], which initially contained three classes—the current version has been expanded to twelve classes. The methodology used for generating the “Top-View Data”, “MultiHeightView Data” and “Depth Data” is described here. The datasets were annotated using Roboflow [9] and CVAT [10].

### 4.1 Real-world data acquisition

Real-world traffic scene data were collected using three primary devices to ensure diverse perspectives and comprehensive data coverage.

The first device, a COOAU D30 Dash Camera, was mounted in a vehicle to capture videos for RealStreet and SupporterReal datasets, covering urban traffic scenes. Installed inside a moving vehicle, the camera provided continuous coverage of urban intersections and road segments. The camera’s fixed-focus ultra-wide optic covers roughly 170° of the scenes, paired with a 1/2.9-inch CMOS image sensor engineered for strong low-light sensitivity and wide dynamic range performance. Video was recorded in full-HD (1920 × 1080) format at 30 frames per second, with the lens maintained in a level orientation (0° pitch) for consistent perspective. Footage was stored in a 16:9 aspect ratio, preserving the wide capture span.

Fisheye-type curvature from the lens was left uncorrected to preserve the raw spatial characteristics of the data. This ensured consistency with real-world conditions, allowing detection and tracking models to operate under the same distortions typical of wide-angle or embedded vision systems.

The second device, an iPhone 15 Pro Max, was used to capture RealHeightView data by recording scenes at three fixed heights (0.5 m, 1.0 m, and 1.5 m) using the main camera. The focal length was 24.00 mm, optimized for capturing clear and detailed images of objects at varying distances. This setup ensured that the captured images free from distortions that could affect depth prediction.

The third device, an Intel RealSense D457 depth-sensing camera, was used to capture RealDepth data with synchronized RGB frames and sensor-derived pixel-wise depth maps. Utilizing its active stereo module, the device provides depth aligned with RGB images. Each scene was recorded from a tripod at a height of 1.5 meters and captured from three angular viewpoints: 0° (center-facing), +30° (rotated right), and –30° (rotated left). RGB and depth resolution were fixed by 1280 × 720 at 30 FPS.The Intel RealSense D457 is factory-calibrated with accurate intrinsic parameters for both RGB and depth sensors. These intrinsics remain valid across recording sessions and varying view angles, as the camera was tripod-mounted at 1.5 m and rotated around a fixed origin. Since the internal geometry and lens characteristics do not change with such rotations, recalibration was unnecessary. This ensured consistent and reliable alignment between RGB and depth data across all viewpoints. The intrinsic parameters, extracted from the camera’s factory calibration, are included in the dataset’s “calib.txt” file to support reprojection and 3D reconstruction. This makes the RealSense D457 practical for multi-angle data collection in real-world environments, where frequent recalibration is impractical.

RealTop was collected in compliance with Japan’s urban UAV regulations, which limit drone-based filming in populated areas. To emulate aerial imagery, recordings were made from elevated positions such as the rooftops of tall buildings. For each capture session, viewing orientation was varied extensively tilts ranged from −60° to 60°, and the camera’s horizontal sweep covered the full 360°. Positioned on elevated structures about 20–30 m high, the camera recorded at 30 frames per second. To further diversify training data, we applied augmentation such as positive/negative rotation and 180° inversion.

To ensure privacy and comply with ethical standards, the real datasets have undergone full-frame anonymization using the following method:

• Full-Frame Gaussian Blur: a Gaussian blur with a specific sigma value 8.0 was applied to the entire video. This ensures that faces, vehicle license plates, and other identifiable details are completely unrecognizable when the video is played.
• Privacy and Ethical Compliance: The dataset complies with privacy regulations and ethical research guidelines by eliminating personally identifiable information (PII). The anonymized dataset is intended solely for research purposes, such as traffic scene analysis and computer vision applications.

### 4.2 Synthetic Data Generation

The creation of synthetic datasets follows a structured process adapted from our earlier work [1, 8] and extended to multiple dataset categories. The workflow begins by analyzing representative frames from real-world recordings to guide scene modeling. Intersection geometry and building footprints are aligned with geographic data from OpenStreetMap, then imported into Lumion for detailed modeling. In our earlier work [1], this workflow was specifically designed for the creation of the SynthStreet dataset, focusing on synthetic horizontal-view traffic scenes. In the current study, the framework has been extended and generalized to support the generation of multiple synthetic datasets [1].

The step-by-step process for synthetic data generation is outlined below:

1. Extracting Real-World Images: Frames were extracted from real video footage to capture authentic traffic scenes.
2. In the content analysis phase, images are processed to identify and segment key elements, such as roads, vehicles, pedestrians, and other environmental features.
3. 3D Model Development: The analysed content was converted into detailed 3D models representing the scene’s structure and objects, as illustrated in (Fig. 7).

**Fig. 7:**
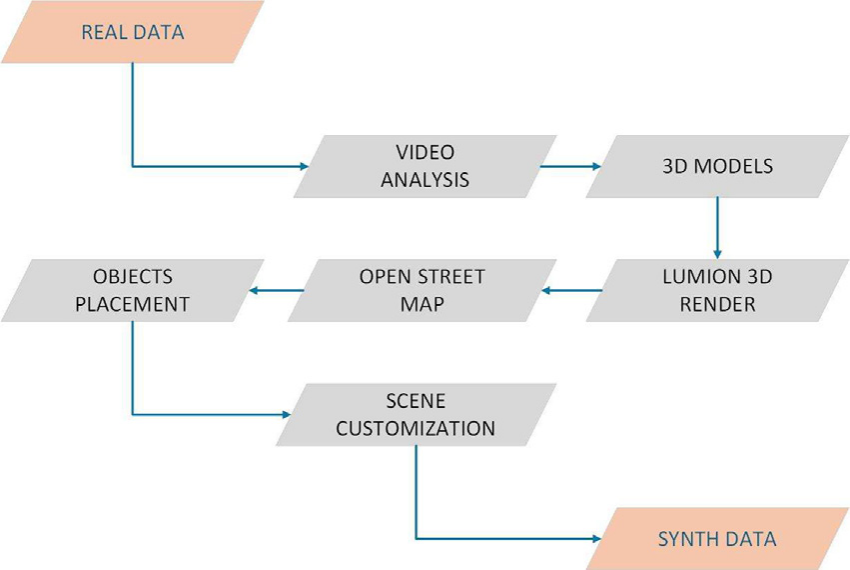
Generalized synthetic data creation pipeline, extended from our previous workflow [1].
4. The integration of 3D models into Lumion using OpenStreetMap data (See Fig. 8) acquiring geographical data for the urban area. OpenStreetMap (OSM) [11] data for the specific intersection area was downloaded in. osm format, providing detailed information about the roads, buildings, and infrastructure in the selected region. This data was used to accurately represent the real-world environment in the simulation. Using ArchiCAD’s tools, the OSM data was transformed into detailed 3D models.

**Fig. 8:**
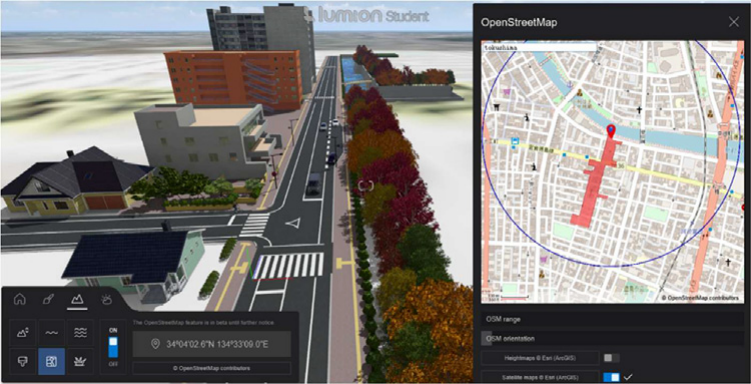
Incorporating 3D models into Lumion scenes using OSM geographic layers, adapted from [1].
5. The imported models were accurately placed to reflect the intersection’s real-world layout.
6. To create a comprehensive and realistic traffic simulation, various environmental factors, including camera height, view angle, and other specific elements, were adjusted to customize the scene.
7. Finally, after the 3D models were fully integrated, the scene was rendered using Lumion’s high-quality rendering capabilities. This process produced high-resolution images and video sequences of the intersection, which can be used for traffic analysis or visualization.

To replicate the real-world traffic scenes in a controlled synthetic environment, we configured Lumion with camera settings that matched the key parameters of our real recordings—including camera height, field of view, and viewing angles. Although an exact match was not feasible due to platform constraints, key camera parameters were carefully aligned to enable meaningful visual and geometric comparisons. Environmental parameters such as weather and lighting are configured to create diverse scenarios. The collection method described here extends the earlier overview in [1] by providing a more comprehensive, parameter-level description.

For the horizontal-view synthetic scenes replicating the Traffic Scene dataset, the virtual camera in Lumion was configured to reproduce the optical characteristics of the real-world capture device. A focal length of 15 mm was selected to approximate the ultra-wide perspective of the COOAU D30 dash camera, which uses a 170° lens. The camera was positioned 1.5 m above the virtual roadway to mirror the viewpoint of an in-vehicle mount. Orientation settings included a heading of –180° to align with the flow of traffic and a pitch of 0.0° to maintain a level view parallel to the road surface. Output was rendered in Full HD (1920 × 1080) with a 16:9 aspect ratio, ensuring visual consistency with the real camera footage.

In this release, SynthTop scenes were designed to closely mirror real-world camera configurations, including matching focal lengths and viewpoints used in actual surveillance setups. In the simulated Lumion environment, the virtual camera was configured to reproduce the geometry of real-world top-view captures.

The camera height was set to 20 m with a focal length of 15 mm, and its tilt varied from −60° to 60° while sweeping horizontally through the full 360°. Output sequences were rendered at 30 frames per second. To broaden appearance diversity, positive and negative rotations and 180° inversions were applied to the rendered images. Compared to its earlier version in [8], SynthTop includes refined annotations, and a dedicated evaluation set.

To replicate the RealHeightView in a synthetic environment, Lumion’s virtual camera was configured to match the three real-world tripod heights: 0.5 m, 1.0 m, and 1.5 m. For each height, the camera was oriented with a heading of –180° and a pitch of 0.0°, maintaining a consistent horizontal forward-facing perspective. The focal length was set to 24.00 mm to approximate the wide-angle coverage of the real iPhone device, and the aspect ratio was configured to 16:9, matching the 1080p resolution of the real recordings.

This setup enabled the creation of synthetic counterparts to the real scenes, allowing for controlled experiments on how monocular depth models respond to variations in camera elevation.

### 4.3 Perceptual similarity evaluation between real and synthetic scenes

To quantitatively assess the perceptual realism of the synthetic scenes relative to the real-world video frames, we reference the Learned Perceptual Image Patch Similarity (LPIPS) metric [12]. LPIPS evaluates the perceptual distance between two images using deep features from pretrained neural networks, offering a more human-aligned measurement of visual similarity than pixel-wise metrics such as SSIM or PSNR.

We evaluated the similarity between real and synthetic frames using a local GPU-based setup with the LPIPS PyTorch implementation. Each video pair was compared at the 3-second mark using spatially aligned frames resized to 256 × 256 resolution and normalized to [-1, 1]. Table 1 shows the observed LPIPS scores ranged from 0.182 to 0.289 across the scenes.

**Table 1:** LPIPS score of synthetic-to-real pairs.

	LPIPS Score
Traffic Scene Data	
Scenario_01	0.211
Scenario_02	0.182
Scenario_03	0.248
Scenario_04	0.193
TopView Data	
Scenario_01	0.289
MultiHeight View Data	
Scenario_0.5m	0.257
Scenario_1.0m	0.271
Scenario_1.5m	0.215
**Average LPIPS Score**	**0.233**

These values indicate moderate-to-high perceptual similarity, consistent with prior literature that considers LPIPS values below 0.30 as indicative of sufficient visual realism for synthetic-to-real domain transfer tasks.

These findings support the conclusion that synthetic scenes exhibit a comparable visual structure to real scenes, both in terms of layout and appearance. The LPIPS results confirm that the synthetic data is visually realistic enough to support this purpose.

### 4.4 Dataset annotation

Traffic Scene dataset employs two parallel annotation strategies tailored to different perception tasks:

Object detection focuses on 3 key classes in real traffic scenes: ‘*car’, ‘cyclist’,* and ‘*person’*. These represent the most critical dynamic agents in real-world traffic scenarios, particularly in the context of Vulnerable Road Users (VRUs) and collision avoidance. Unlike static elements, these classes require precise localization and tracking for safety-critical applications. This distinction is intentional and reflects the functional difference between segmentation and detection tasks. In traffic scenes, a person riding a bicycle is functionally one agent of a cyclist. Treating them as separate objects (as in COCO: ``person'' + "bicycle") requires extra logic to associate them, which is error-prone and computationally inefficient.

We define the cyclist as a unified class to better represent its functional role in traffic. As a view of VRUs recognition, cyclists, like pedestrians, are classified as Vulnerable Road Users (VRUs), just like pedestrians. From a safety and behavior analysis perspective, detecting them as a single class is more meaningful and aligned with real-world needs.

In contrast, semantic segmentation includes 12 classes in synthetic scenes: ‘car’, ‘person’, ‘cyclist’, ‘road’, ‘pavement’, ‘background’, ‘building’, ‘lane’, ‘zebra’, ‘green’, ‘traffic_light’, ‘stone’, and ‘Others’. These classes capture both dynamic agents and static infrastructure commonly found in urban environments. Notably, the green class combines trees, grass, and other vegetation into a unified category to simplify annotation and avoid unnecessary semantic fragmentation, while still providing relevant contextual information for scene understanding. For object detection tasks, we excluded the ‘background’ and ‘Others’ classes, resulting in 11 refined detection classes: building, car, cyclist, green, lane, pavement, person, road, stone, traffic_light, and zebra.The number of annotated instances is shown in Table 2 reflects the total number of objects labeled specifically for object detection tasks, using the YOLO format (class_id x_center y_center width height) as the annotation standard. The Traffic Scene and TopView datasets include a total of 101,245 annotated objects, distributed across three core traffic participant classes. ‘Car’ class constitute the largest portion with 50,228 instances (49.61%), followed by ‘person’ class with 31,786 (31.40%), and ‘cyclist’ class with 19,231 (18.99%). This distribution reflects the typical composition of urban traffic and supports class-balanced evaluation of detection and segmentation models.

**Table 2:** Class and object ratio in detection.

Class ID	Class name	Object Count	Images Containing
0	car	50228	7852
1	cyclist	19231	6479
2	person	31786	6998

Table 3 shows that a total of 15,090 segmentation masks annotated across thirteen classes in Traffic Scene. These masks are formatted in COCO-style JSON. The class distribution is as follows: car (6,185 instances), cyclist (3,630), person (2,170), green (1,145), and traffic_light (2,960). These categories were chosen to reflect common road user types and environmental features critical for autonomous driving and traffic monitoring tasks.

**Table 3:** Class and object ratio in segmentation.

	Class Name	Segmentation Count
1	car	6185
2	cyclist	3630
3	person	2170
4	green	1145
5	traffic_light	2960
6	zebra	485
7	lane	5070
8	road	3455
9	pavement	2570
10	stone	3865
11	Other	4840
12	background	95
13	building	3875

## Limitations

While this dataset provides a unified collection of real and synthetic traffic scenes across multiple viewpoints, it has certain limitations. The data were collected within a limited geographic area in Tokushima, Japan, which may affect the generalizability of models trained solely on this dataset to other urban environments with different traffic behaviors and infrastructure layouts. Additionally, the real-world recordings rely on monocular RGB cameras and stereo depth sensors, without multi-sensor data such as LiDAR.

This dataset was developed by a small research team and does not aim to match the scale or multimodal complexity of large public benchmarks such as KITTI, VKITTI, or nuScenes. Instead, the dataset is designed to address a distinct use case: the augmentation of limited real-world data with photorealistic synthetic content in privacy-constrained or resource-limited environments.

While the dataset may have limitations in terms of geographic diversity, volume, and sensor variety, it provides aligned real and synthetic traffic scenes captured from consistent viewpoints. This structure enables controlled experimentation for tasks such as domain adaptation, and perception model training in urban traffic contexts. Future expansions will include additional scenarios, sensors, and environmental conditions to further enhance dataset diversity. Future versions aim to expand geographic coverage, increase scenario diversity, and integrate additional sensor modalities to enhance its representativeness and utility.

## Ethics Statement

The authors confirm that they have read and follow the ethical requirements for publication in Data in Brief. To ensure privacy and comply with ethical standards, all real datasets have undergone full-frame anonymization using Gaussian blur, effectively removing personally identifiable information (PII). The dataset is intended solely for research purposes, such as traffic scene analysis and computer vision applications.

## Credit Author Statement

**Khulan Khalzaa:** Methodology, Writing – original draft, Writing - Review & Editing, Data curation, Visualization; **Stephen Karungaru:** Supervision, Methodology, Writing - Review & Editing, Resources; **Kenji Terada:** Methodology, Writing - Review & Editing, Resources; **Tsend-Ayush Chimed-Ochir:** Writing - Review & Editing.

## Data Availability

Mendeley DataPhoto-Realistic Traffic Scene Datasets: Real and Synthetic Perspectives (Original data).
